# Cyclin-dependent kinase 6 (CDK6) as a potent regulator of the ovarian primordial-to-primary follicle transition

**DOI:** 10.3389/fcell.2022.1036917

**Published:** 2022-12-23

**Authors:** S. Ataei-Nazari, M. Amoushahi, JF. Madsen, J. Jensen, A. Heuck, A. Mohammadi-Sangcheshmeh, K. Lykke-Hartmann

**Affiliations:** ^1^ Department of Biomedicine, Aarhus University, Aarhus, Denmark; ^2^ Department of Animal Science, University of Tehran, Tehran, Iran; ^3^ Department of Clinical Genetics, Aarhus University Hospital, Aarhus, Denmark

**Keywords:** primordial follicle activation, CDK, oocytes, ovary, *in vitro*

## Abstract

**Introduction:** Ovarian follicle development requires tight coordination between several factors to initiate folliculogenesis to generate a mature and fertile egg. Studies have shown that cell cycle factors might contribute to follicle development, hover specific knowledge on individual CDKs and follicle activation has not been investigated. Among cell cycle regulators, *CDK6* is a key player through binding to cyclin D resulting DNA synthesis and genome duplication. Interestingly, the *CDK6* gene is differentially expressed in oocytes and granulosa cells from human primordial and primary follicles, which suggest a potential role of *CDK6* in the primordial-to-primary transition. In this study, we investigated the potential regulatory role of *CDK6* in progression of primordial to primary follicle transition using BSJ-03-123 (BSJ), a CDK6-specific degrader.

**Methods:** In mouse ovarian *in vitro* culture, BSJ reduced the activation of primordial follicles, and reduced follicle development. As a next step, we examined the egg maturation read-out and found that BSJ-treated follicles matured to competent MII eggs with resumption of first meiosis, comparable with the control group.

**Results:** Noteworthy, it appears that inhibition of *CDK6* did increase number of apotoptic cells, articular in the granulosa cells, but had no impact on ROS level of cultured ovaries compared to control group, indicating that the cells were not stressed. Oocyte quality thus appeared safe.

**Discussion:** The results of this study indicate that *CDK6* plays a role in the primordial-to-primary transition, suggesting that cell cycle regulation is an essential part of ovarian follicle development.

## Introduction

The cell cycle progression is a vital and highly preserved physiologic process, which controls the genome duplication (Ding et al., 2020). In mammals, a highly stage-managed series of mitotic and meiotic cell cycles is required for folliculogenesis and oogenesis. During gonadal development, oogonia begins prophase of the first meiotic division and proceed through leptotene, zygotene and pachytene stages, followed by arresting in diplotene stage to become oocyte. The oocyte stays in the diplotene stage and is surrounded by a single layer of flattened pre-granulosa cells, forming dormant (primordial) follicles. In a process known as primordial follicle activation, a cohort of primordial follicles is activated into primary follicle stages (Persson et al., 2005), a follicle stage characterized by an oocyte surrounded by a single layer of cubical granulosa cells. Granulosa cells progress dynamic control of mitotic cell cycle process during folliculogenesis. At the end of follicle development, the mature oocyte arrest after the first meiotic division. Both meiotic and mitotic cell cycle processes are developmentally regulated and playing important role during folliculogenesis and oogenesis (Persson et al., 2005). The cell cycle process is governed by several cyclins and cyclin-dependent serine/threonine kinases (CDKs) (Ding et al., 2020).

CDKs include over 20 members and with their sequential activation and phosphorylation, in association with D-type cyclins, regulate the exit from G1 to S phase in cell cycle (Tigan et al., 2016). Among CDKs, CDK6, along with its partner CDK4, are key players in cell cycle progression through binding to cyclin Ds resulting phosphorylation of the retinoblastoma protein (Rbp), and inducing DNA synthesis. One targeted therapy for CDK inhibition is palbociclib, which may slow the growth of advanced stage breast cancers. In line with this, Palbociclib was used to protect chemin-induced human granulosa cells from apoptosis, acting to inhibit the p53/p21 pathway (Li et al., 2019).

The activity of CDK4 and CDK6 is controlled by p16, whereas p27 bind to a broad range of CDK-cyclin complex. Previous reports showed p27 to be a suppressor of ovarian follicle endowment/formation and activation, and an enhancer of ovarian follicle atresia (Jirawatnotai et al., 2003). On the other hand, the early oocyte and follicle growth was coincided with reduction in p16 expression demonstrating the relationship between CDK6 and follicle development (Bayrak and Oktay, 2003). In line with this, the Cyclin dependent kinase inhibitor 1B (CDKN1B) controlled ovarian development in mice by suppressing follicle endowment and activation, and also promoting follicle death (Rajareddy et al., 2007), comparable to p27. CDKN1B controls the activation of cyclin CDK6/4-cyclin D complexes, and thus governs the cell cycle progression at G1 (Nebenfuehr et al., 2020). In a recent study conducted in porcine oocytes, inhibition of CDK2, CDK4, and CDK6 was not influential on cumulus expansion or germinal vesicle breakdown, whereas CDK7 and CDK9 inhibition reduced germinal vesicle breakdown and cumulus expansion (Oqani et al., 2017).

Previous data revealed that the CDK6 gene was expressed in human primordial and primary follicles, where transcriptional levels of *CDK6* showed downregulation in oocytes and upregulation in granulosa cells, from primordial to primary follicle transition in human ovarian tissue (Ernst et al., 2017; Ernst et al., 2018).

In order to determine the possible role of CDK6 in the primordial-to-primary transition, BSJ-03-123 (BSJ), a degrader with proteome-wide selectivity for CDK6, which uniquely enables rapid pharmacological interrogation of CDK6- dependent functions, was used. To address the role of CDK6 in follicle development. BSJ was added to primary ovary organ culture to assess the effect *in vitro*. Interestingly, BSJ inhibited primordial follicle activation in a concentration-dependent manner, aligned with a reduced level of CDK6 protein, suggested the possible role of CDK6 in primordial follicle regulation. Moreover, the ability of the BSJ-treated follicles to mature to MII oocytes was revealed, suggesting BSJ as a future clinical candidate for regulating primordial follicle activation, eg., during chemotherapy, to prevent premature ovarian exhaustion.

## Results

### *CDK6* was present in human primordial and primary follicles

In the present study, we extracted the *CDK6* gene expression from transcriptomic analysis studies applied in isolated human oocytes and granulosa cells from primordial and primary follicles (Ernst et al., 2017; Ernst et al., 2018).

**FIGURE 1:**
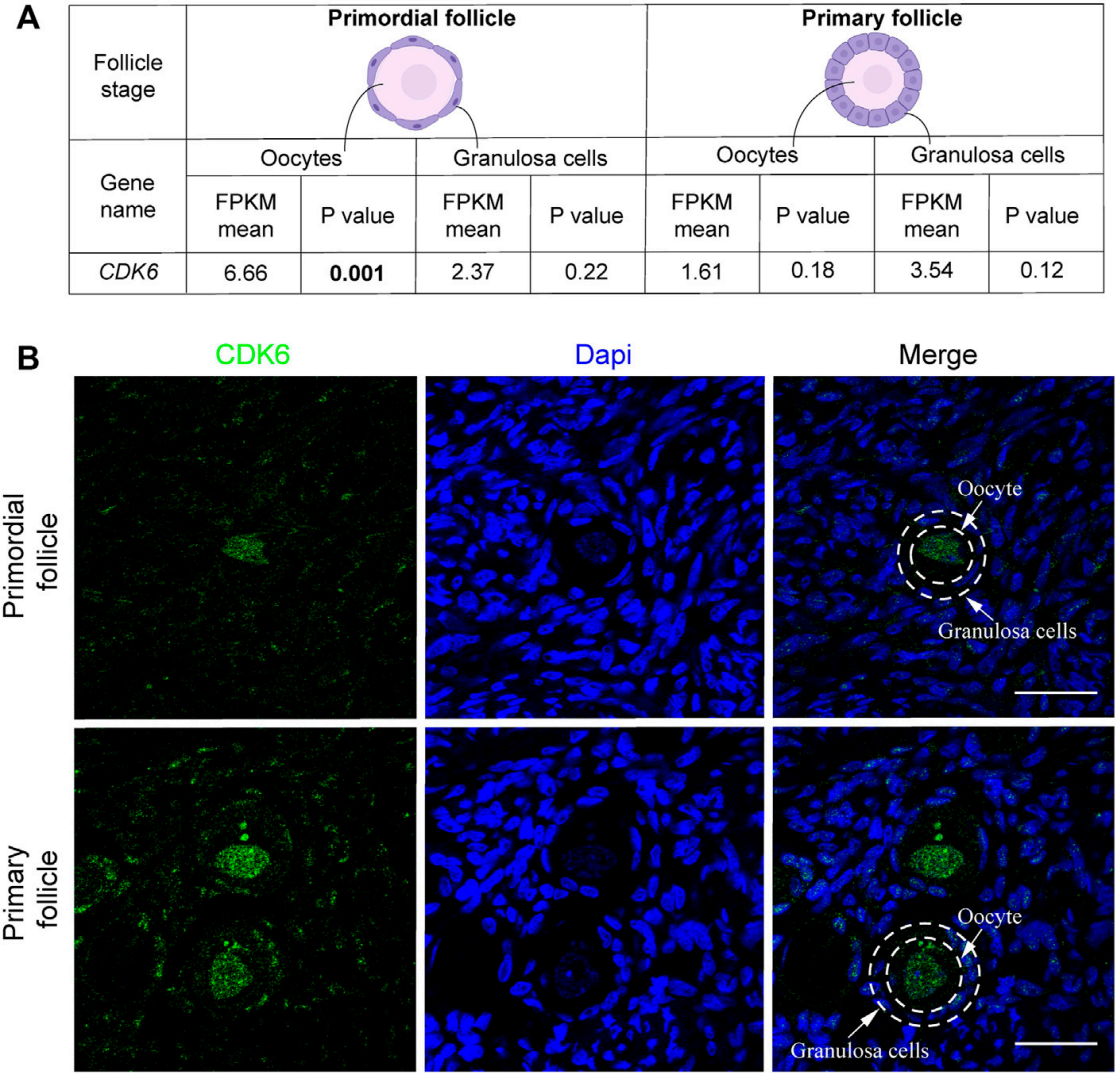
CDK6 mRNA and protein expression in human ovarian tissue. **(A)** Table with FPKM and *p* values of the gene expression of *CDK6* mRNA in human oocytes and granulosa cells of the primordial and primary follicle, and illustration of the morphology of the follicular stages. Significant *p*-values are noted in bold. **(B)** CDK6 protein expression in preantral follicle stages in human tissue. Negative controls are shown in Supplementary Figure S1. Scale bar: 20 µm.

Interestingly, transcriptional level of *CDK6* was downregulated from 6.66 to 1.61 fragments per kilobase of exon model per million reads mapped (FPKM) (mean) in oocytes from primordial to primary follicle transition (Figure 1A). In contrast, *CDK6* expression was upregulated in granulosa cells from 2.37 to 3.54 FPKM from primordial to primary follicle activation (Figure 1A). In summary, as the *CKD6* transcript is reduced in human oocytes from the primordial-to-primary transition, the *CDK6* transcript is slightly upregulated in the granulosa cells. In the same transition, suggesting an active role of CDK6. To interrogate the CDK6 protein expressed, immunofluorescence analysis on human ovarian tissue using a CDK6 antibody was performed. The Immunofluorescence results showed the presence of the CDK6 protein in both oocytes and granulosa cells of follicles at different developmental stages. Interestingly, a predominant nuclear distribution of CDK6 protein in the oocytes from both primordial and primary follicles was noted (Figure 1B). A no-primary-antibody negative control detected no specific staining (Supplementary Figure S1A).

### BSJ reduced the level of CDK6 protein in mouse ovaries

**FIGURE 2:**
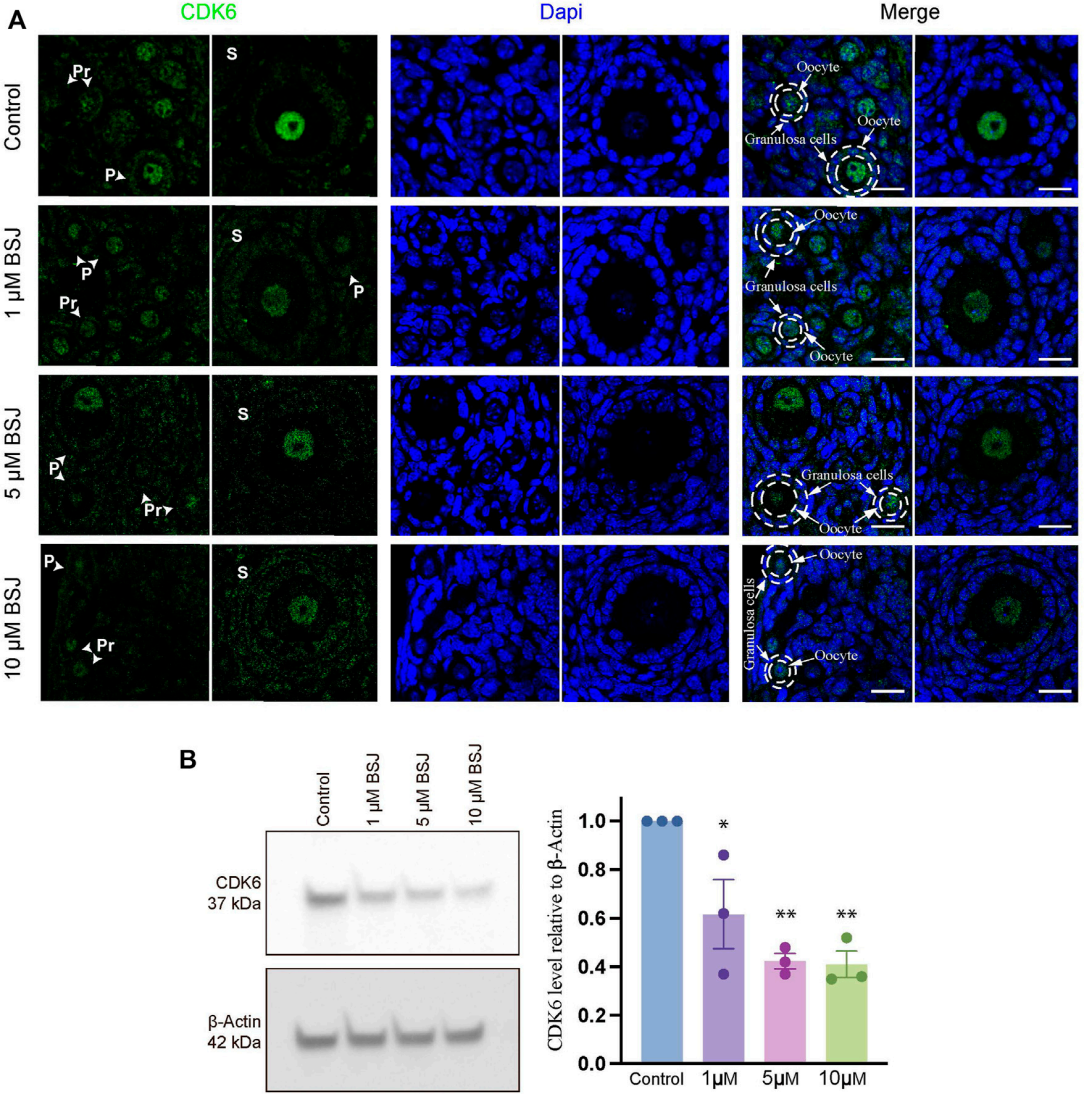
Nuclear expression of CDK6 in murine ovarian follicles after 5 days *in vitro* culture with 0–10 µM BSJ. **(A)** Immunofluorescent staining of CDK6 and counterstained with DAPI in primordial (Pr), primary (P) and secondary (S) Scalebar: 20 µm. Negative controls are represented in Supplementary Figure S1. **(B)** Representative western blotting with primary antibodies against CDK6 and beta-actin as loading control on ovaries *in vitro* cultured with different concentrations of BSJ (0–10 µM) for 5 days. Full-length membranes are represented in Supplementary Figure S4. Data reflects three independent biological replicates, *n = 12,* and are represented as mean ± SEM. Data are analyzed with one-way ANOVA followed by Bonferroni correction where the mean of each concentration is compared with the mean of the control. Statistically significant data are noted with asterisks, *p < .05, **p < .01.

To evaluate the role of CDK6 on primordial follicle activation, a pharmacological approach was applied using BSJ, as a selective CDK6 degrader. As a first indication of the significance of CDK6 in mouse ovarian tissue, immunofluorescence staining was performed on mouse *in vitro* cultured ovaries with or without BSJ at 1, 5, and 10 µM concentrations using an anti-CDK6 antibody. Likewise immunofluorescence staining in human ovarian tissue, CDK6 protein was distributed in both oocytes and granulosa cells, particularly in nuclei of oocytes, from follicles at primordial, primary and secondary follicle developmental stages (Figure 2A). Moreover, the expression of CDK6 protein in follicles at different developmental stages in cultured ovaries with BSJ was reduced compared to DMSO control group, as expected (Figure 2A). Immunofluorescence on mouse ovarian tissue without the primary antibody was shown (Supplementary Figure S1B) and detected no specific staining. Next, Western blotting analysis was applied to evaluate whether the expression of CDK6 protein was likewise decreased in mouse *in vitro* cultured ovaries with BSJ at 1, 5, and 10 µM concentrations compared to DMSO control groups. As expected, the results demonstrated that CDK6 protein level from *in vitro* cultured ovaries with BSJ were significantly lower than DMSO control group (Figure 2B; Supplementary Figure S4) (Table 1).

**TABLE 1:** Western blotting data.

Bonferroni multiple comparison test	Adjusted *p*-value	Summary
Control vs. 1 μM	.0244	*
Control vs. 5 μM	.0023	**
Control vs. 10 μM	.0020	**

Statistically significant data are noted with asterisks, *p < .05, **p < .01, ***p < .001, ****p < .0001, ns, not significant.

### Reduction of CDK6 reduced primordial follicle activation in mouse ovaries

**FIGURE 3:**
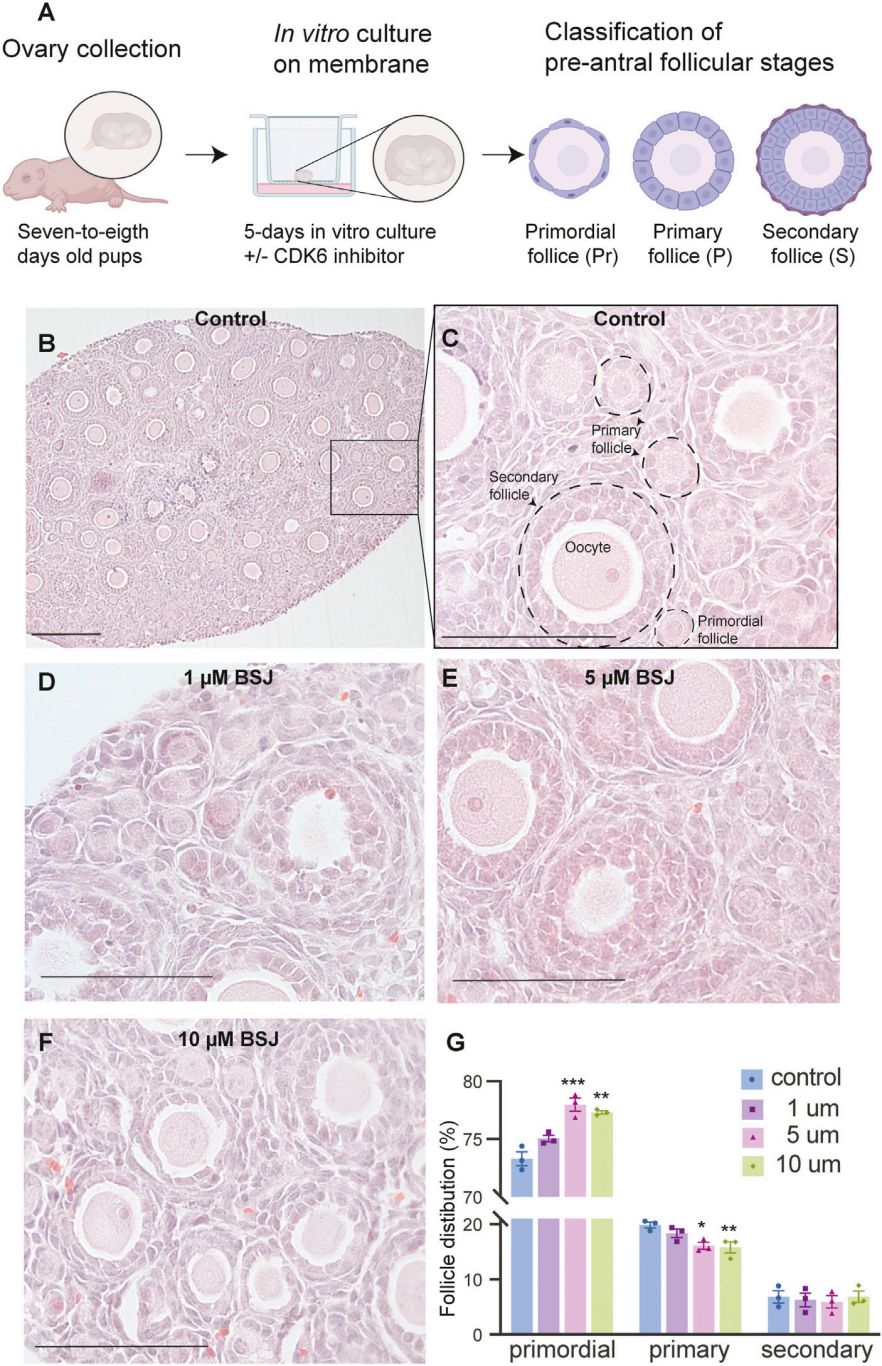
Inhibition of CDK6 suppress primordial follicle activation *in vitro.* **(A)** Schematic illustration of the experimental design and morphological characteristics of primordial (Pr), primary (P) and secondary (S) follicles. **(B)** Photomicrograph of whole ovary cultured 5 days *in vitro* in control medium. Scale bar: 100 µm. **(C)** The square defined in B is examined more closely to assess the follicular morphology, and follicular structures are defined. Scale bar: 50 µm. **(D–F)** Representative photomicrograph of H&E-stained ovary exposed to 1 µM BSJ **(D)**, 5 µM BSJ **(E),** and 10 µM BSJ **(F)**. Scalebar: 50 µm. Photomicrographs of whole ovaries and more representative sections of the ovary are shown in Supplementary Figure S2. **(G)** The distribution of primordial, primary and secondary follicles in ovaries exposed to 0–10 µM BSJ. Data are also represented in table 1 and includes 30–40 µM of BSJ, and photomicrographs of H&E-stained ovaries of these concentrations are shown in Supplementary Figure S2. For all concentration *n* = 3. The data is analyzed with one-way ANOVA followed by Bonferroni correction where the mean of each concentration is compared with the mean of the control. Statistically significant data are noted with asterisks, *p < .05, **p < .01, ***p < .001, ****p < .0001.

From above, it was evident that BSJ reduced levels of the CDK6 protein in ovaries. To functionally test of CDK6 inhibition would alternate follicle distribution *in vitro*, neonate ovaries were cultured on inserts for 5 days without or with BSJ at different concentrations (Figure 3A). Follicles were classified as primordial, primary and secondary follicles, as illustrated (Figure 3A). The morphology of granulosa cells and oocytes were persistent and follicles at different developmental stages were well organized in cultured ovaries at 1 and 5 µM concentrations of BSJ and DMSO control groups (Figures 3B–F). However, the shape of oocytes in growing follicles at 10–40 µM concentration of BSJ were irregular and less organized compared to the DMSO control group (Supplementary Figures S2, S3A). Interestingly, BSJ at 5, 10, 20, 30, and 40 µM concentrations significantly increased the percentage of primordial follicles (77.97 ± 0.58, 77.30 ± 0.14, 78.64 ± 0.17, 79.91 ± 0.80, and 80.21 ± 1.06) compared to DMSO control group (73.30 ± 0.59), while the percentage of primary follicles were significantly decreased in cultured ovaries with these concentrations (16.10 ± 0.62, 15.81 ± 1.01, 13.57 ± 0.23, 12.00 ± 0.50, and 11.89 ± 1.04) compared to DMSO control group (19.87 ± 0.53) (Figure 3G; Supplementary Figure S3B) (Table 2, Table 3, Table 4, and Table 5). The most significant effect of BSJ was observed at 5 µM (Figure 3G) However, the percentage of secondary follicles were not significantly changed among groups (Figure 3G; Supplementary Figure S3B) (Table 2, Table 3, Table 4, and Table 5).

**TABLE 2:** Summary of follicle distribution in ovaries in vitro cultured with 0-40 µM BSJ. Data are represented as mean ± SEM. For all groups *n*=3. Data are analyzed with one-way ANOVA followed by Bonferroni correction. H&E images of whole and representative sections of the ovaries is shown in Supplementary Figures S2,S3.

	Control	1 μM	5 μM	10 μM	20 μM	30 μM	40 μM	*p*-value
Primordial follicle (%)	73.30 ± 0.59	75.04 ± 0.29	77.97 ± 0.58	77.30 ± .14	78.64 ± .17	79.91 ± 0.80	80.21±1.06	< .0001
Primary follicle (%)	19.87 ± 0.53	18.35 ± .078	16.10 ± 0.62	15.81 ± 1.01	13.57 ± .23	12.00 ± 0.50	11.89±1.04	< .0001
Secondary follicle (%)	6.84 ± 1.10	6.29 ± 1.25	5.94 ± 1.15	6.89 ±.03	7.90 ± .18	8.10 ± .30	7.90 ± .28	ns

**TABLE 3:** Summary of multiple comparison tests for primordial follicle.

Bonferroni multiple comparison test	Adjusted p-value	Summary
Control vs. 1 μM	.3712	ns
Control vs. 5 μM	.0005	***
Control vs. 10 μM	.0022	**
Control vs. 20 μM	.0002	***
Control vs. 30 μM	< .0001	****
Control vs. 40 μM	< .0001	****

Statistically significant data are noted with asterisks, *p < .05, **p < .01, ***p < .001, ****p < .0001, ns: not significant.

**TABLE 4:** Summary of multiple comparison tests for primary follicle.

Bonferroni multiple comparison test	Adjusted p-value	Summary
Control vs. 1 μM	.9588	ns
Control vs. 5 μM	.0148	*
Control vs. 10 μM	.0085	**
Control vs. 20 μM	.0002	***
Control vs. 30 μM	< .0001	****
Control vs. 40 μM	< .0001	****

Statistically significant data are noted with asterisks, *p < .05, **p < .01, ***p< .001, ****p < .0001, ns: not significant.

**TABLE 5:** Summary of multiple comparison tests for secondary follicle.

Bonferroni multiple comparison test	Adjusted p-value	Summary
Control vs. 1 μM	>.9999	ns
Control vs. 5 μM	>.9999	ns
Control vs. 10 μM	>.9999	ns
Control vs. 20 μM	>.9999	ns
Control vs. 30 μM	>.9999	ns
Control vs. 40 μM	>.9999	ns

Statistically significant data,*p < .05, **p < .01, ***p < .001, ****p < .0001, ns: not significant.

### BSJ-induced CDK6 reduction enhanced apoptosis and ROS level

**FIGURE 4:**
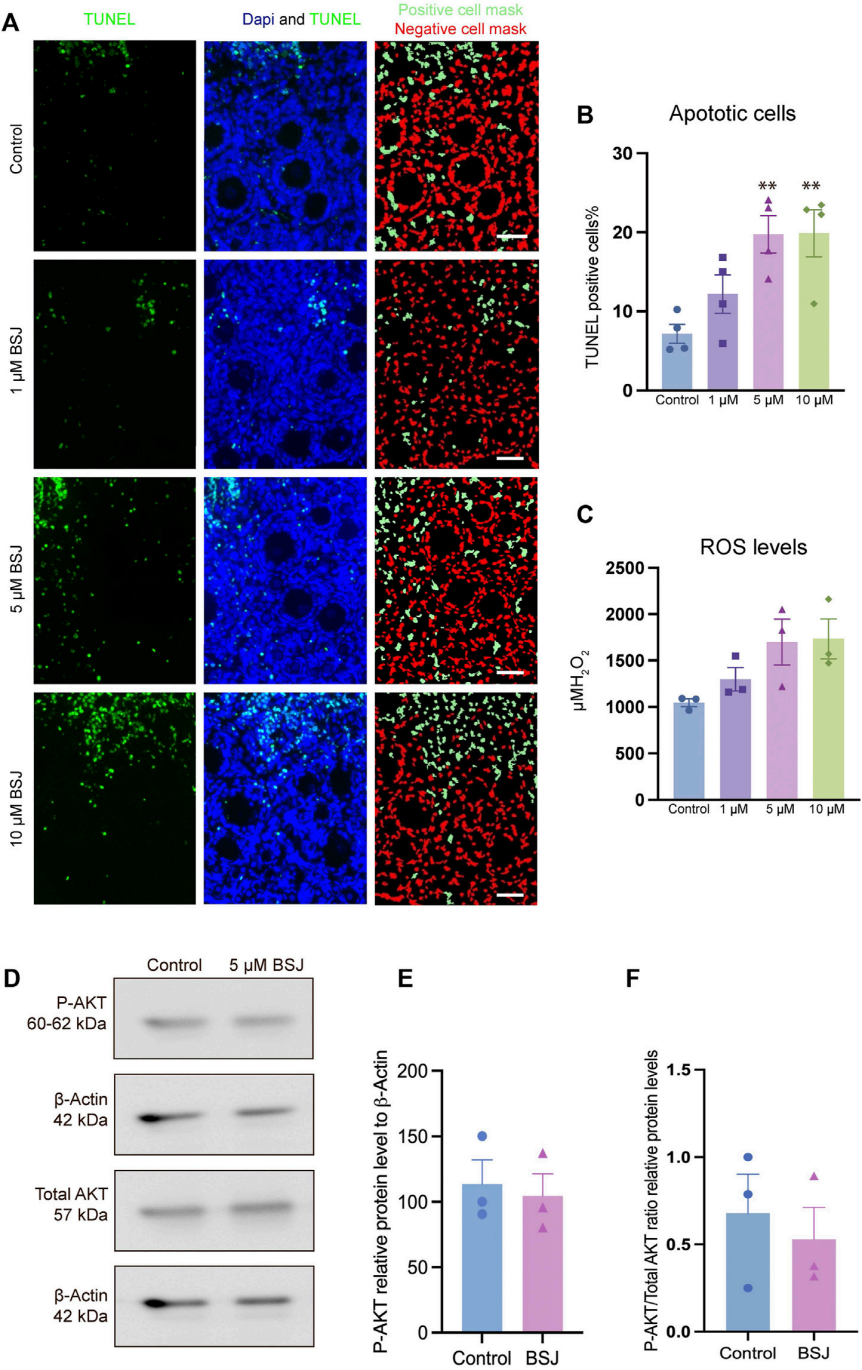
TUNEL and ROS quantification of ovaries cultured with different concentrations of BSJ (0–10 µM) *in vitro* for 5 days. **(A)** The number of apoptotic cells were detected with TUNEL staining. Row 1: TUNEL stain, Row 2: Merge of TUNEL and DAPI stain, row 3: Analysis of apoptosis quantification using an automated cell imaging system. Green cell mask represents cell positive for TUNEL stain, whereas red cell mask represents viable cell mask. Negative controls are represented in Supplementary Figure S1. **(B)** Quantification of percentage of cells positive for TUNEL stain. For all groups *n* = 4. **(C)** Quantification of ROS levels in ovaries *in vitro* cultured for 5 days with different concentration of (0–10 µM) BSJ. For all groups *n* = 6. **(B,C)** Data are analyzed with one-way ANOVA followed by Bonferroni correction where the mean of each concentration is compared with the mean of the control. Statistically significant data are noted with asterisks, *p < .05, **p < 0.01. **(D)** Representative Western blotting with primary antibodies against Phospho-AKT, AKT and beta-actin as loading control on ovaries *in vitro* cultured with 0 and 5 µM BSJ for 5 days. Full-length membranes are represented in Supplementary Figure S4. Data reflects three independent biological replicates, *n = 12* in each group*,* and are represented as mean ± SEM. Data are analyzed with a two-tailed *t*-test.

To evaluate the effect of CDK6 inhibition on apoptosis, TUNEL assay on cultured ovaries with and without BSJ at 1, 5, and 10 µM concentrations was performed. The results demonstrated that particularly, granulosa cells had undergone apoptosis compared to oocytes in all groups (Figure 4A). The proportion of apoptotic cells in cultured ovaries with BSJ at different concentrations increased in a dose dependent manner. Moreover, the proportion of apoptotic cells in cultured ovaries with BSJ at 5 and 10 µM concentrations (19.76 ± 2.35, 19.89 ± 2.98, respectively) was significantly higher than those of cultured in DMSO control group (7.16 ± 1.19) (Figure 4B) (Table 6, and Table 7). Negative control of TUNEL staining with no reacting enzyme on mouse ovarian tissue detected no apoptosis. (Supplementary Figure S1C).

**TABLE 6:** Number of apoptotic cells represented as mean ± SEM. *n*= 4.

	Control	1 μM	5 μM	10 μM
Apoptotic cells (%)	7.165 ± 1.196	12.19 ± 2.421	19.76 ± 2.356	19.89 ± 2.985

**TABLE 7:** TUNEL data.

Bonferroni multiple comparison test	Adjusted p-value	Summary
Control vs. 1 μM	.4604	ns
Control vs. 5 μM	.0073	**
Control vs. 10 μM	.0068	**

Statistically significant data are noted with asterisks, *p < .05, **p < .01, ***p < .001, ****p < .0001, ns, not significant.

In order to assess the effect of CDK6 inhibition on ROS level, as one of the important criteria to define the quality of oocytes and follicles, we investigated ROS levels in mouse *in vitro* cultured ovaries with or without BSJ at 1, 5, and 10 µM concentrations using the 2^′^,7^′^-dichlorodihydrofluorescein diacetate (DCFH-DA) fluorescence assay. As expected, the ROS level in cultured ovaries with BSJ at different concentrations, was increased in a dose dependent manner (Figure 4C). However, the results showed no significant difference among ROS level of cultured ovaries with BSJ at 1 (1298 ± 125.6), 5 (1699 ± 248.5) and 10 (1734 ± 215.7) µM concentrations and DMSO control group (1046 ± 41.37) (Table 8 and Table 9).

**TABLE 8:** ROS data represented as mean ± SEM. *n*= 6.

	Control	1 μM	5 μM	10 μM
ROS levels (μM H2O2)	1046 ± 41.37	1298 ± 125.6	1699 ± 248.5	1734 ± 215.7

**TABLE 9:** ROS data.

Bonferroni multiple comparison test	Adjusted p-value	Summary
Control vs. 1 μM	> .9999	ns
Control vs. 5 μM	.0942	ns
Control vs. 10 μM	.0756	ns

As we observed that BSJ could reduce the activation of primordial follicles, and that cell quality appeared good in regards to apoptosis and ROS levels, a next step was to evaluate if there would be any differences in the classical P-AKT and AKT levels. We observed no differences in P-AKT or AKT in ovaries treated with BSJ compared to the DMSO control (Figures 4D–F), and therefore, it appears that BSJ-treatment does not involve the AKT pathway to detectable levels.

### *In vitro* matured MII oocytes derived from exposed ovaries to BSJ resumed the first meiosis

**FIGURE 5:**
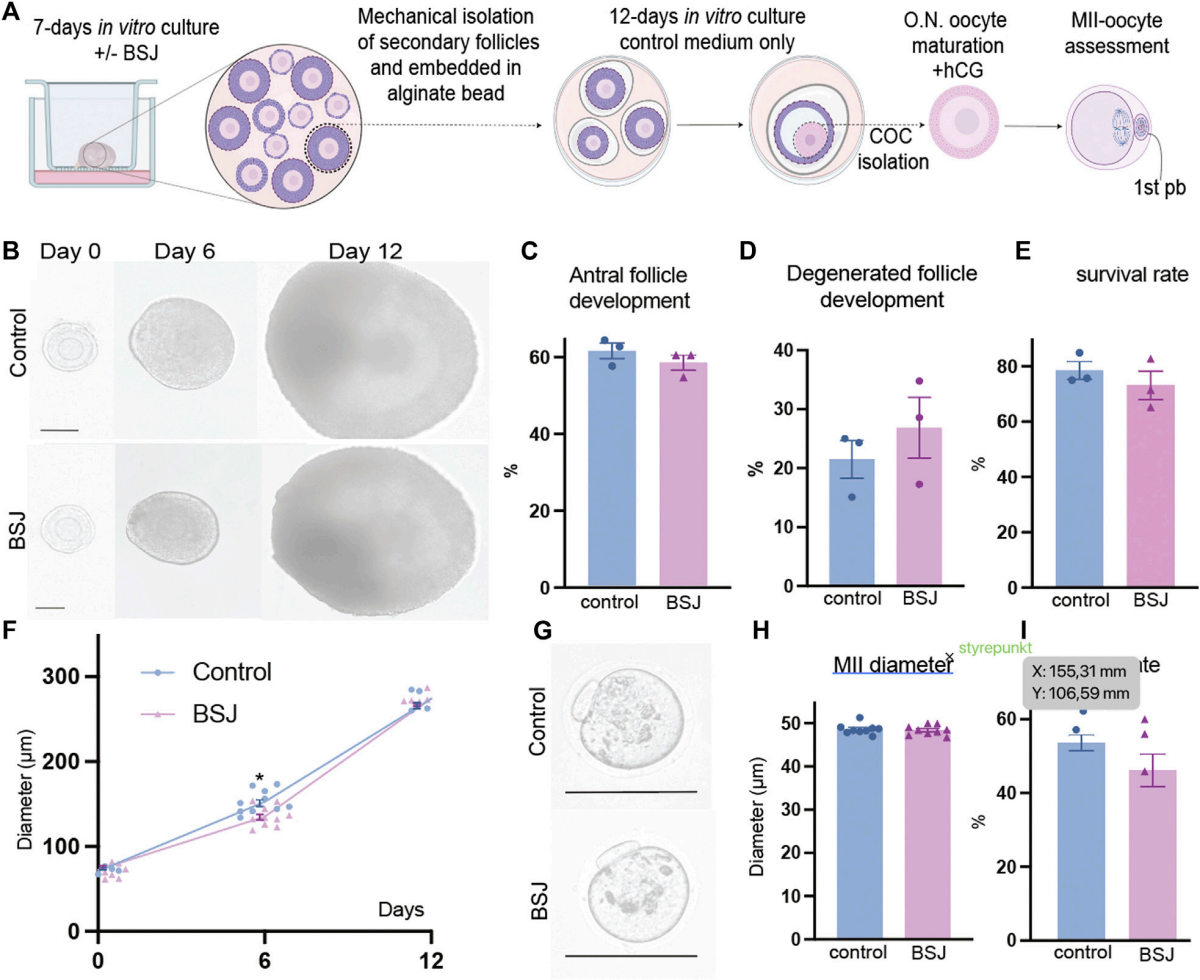
Oocytes exposed to BSJ during primordial follicle activation can still resume the first meiosis. **(A)** Schematic illustration of the experimental design of the *in vitro* strategy for developing MII oocytes from primordial follicles. **(B)** Photomicrographs of follicles during the 12-day three-dimensional follicle culture at day 0, 6, and 12. Scale bar: 50 μm and the respective diameters of the follicles at day 0, 6, and 12. For all groups *n* = 10. **(C)** Antral follicle development (*n* = 22–29). **(D)** Degenerated follicle development (*n* = 12). **(E)** Survival rate (*n = 36–47*). **(F)** Representative photomicrographs of MII oocytes. Scalebar: 50 µm. **(G)** MII diameter, *n = 9.* **(H)** MII rate *n* = 21–29. Data are represented as mean ± SEM and analyzed with a two-tailed *t*-test, *p < .05.

One of the most important criteria to evaluate the quality of oocytes is to assess their meiotic competence. Therefore, we applied a two-step culture system for further analysis of oocyte quality in exposed ovaries to BSJ. First, the ovaries were cultured for 7 days in two groups as BSJ and DMSO control. Then the isolated secondary follicles were cultured in three-dimensional culture system using basic α-MEM (with no BSJ or DMSO) for 12 days, followed by ovulation induction and obtaining MII oocytes (Figure 5A). The morphology of follicles and which were isolated from cultured ovaries in BSJ were comparable to those of isolated from cultured ovaries in DMSO control groups (Figure 5B). Moreover, the secondary follicles in both groups had capability to grow up to antral follicle stage (Figure 5C) and there was no significant difference in the number of survived and degenerated follicles between two groups (Figure 5D, E) (Table 10). To examine and compare the development of follicles, we measured the diameter of follicles during 12 days culture period. The results showed that diameter of follicles in BSJ group at day 6 (135.3 ± 3.72) was significantly lower than that of control group (146.6 ± 2.97), which showed the effect of BSJ on size of follicles. However, the diameter of follicles at day 0 (72.49 ± 1.48) and 12 (274.5 ± 0.59) in BSJ group was not significantly different from those of control group at these days (71.00 ± 2.74 and 273.9 ± 4.24, respectively) (Figure 5F). After *in vitro* oocyte maturation, we showed that morphology of MII oocytes which were derived from cultured ovaries in BSJ were similar to those of derived from cultured ovaries in DMSO control groups, as MII oocytes had regular and round shape with normal polar body in both groups (Figure 5G). In order to analysis the maturation of oocytes, we measured the diameter of *in vitro*-matured MII oocytes and the results showed no significant difference between BSJ (48.40 ± 0.37) and control (48.61 ± 0.39) groups (Figure 5H). Moreover, MII oocyte maturation rate in BSJ group (53.94 ± 4.21) was not significantly different from that of control group (61.08 ± 2.02) (Figure 5I) (Table 10).

**TABLE 10:** Follicle and MII data.

	Control	5 μM	p-value
Antral follicle development	61.08 ± 2.029	58.06 ± 1.944	.3417
Degenerated follicle development	21.47 ± 3.195	26.87 ± 5.135	.4239
Survival rate	75.53 ±3.195	73.13 ± 5.135	.4230
MII diameter	48.61 ± 0.3985	48.40 ± 0.3796	.6984
MII rate	61.08 ± 2.029	53.94 ± 4.217	.2017

## Discussion

In the present study, reduction of CDK6 using BSJ reduced primordial follicle activation, elucidating a role of CDK6 in transition from primordial to primary follicle stage. In our approach, we thought to accomplish a reduction of CDK6 activity, rather than a full inhibition, which was still very effective to reduce the activation of primordial follicles. It has been shown that reduction of CDK6 and cyclin D1 expression prevented proliferation of primordial germ cells in the porcine ovary due to inhibition of G1/S transition resulting in cell cycle arrest (Dai et al., 2021). Beside the role of CDK6 in cell cycle progression, CDK6 is also important in cell cycle independent functions (Malumbres et al., 2004; Hu et al., 2009; Hu et al., 2011; Laurenti et al., 2015; Scheicher et al., 2015). CDK6 can enhance vascular endothelial growth factor (VEGF) and angiogenesis (Kollmann et al., 2013). Recent studies showed that VEGF could activate primordial follicles through formation of blood vessels and induce angiogenesis (Nilsson et al., 2007; McFee et al., 2012; Komatsu and Masubuchi, 2020). Although the molecular mechanisms regulating primordial follicle activation are still being ambiguous, many studies have shown that PI3K/AKT signaling pathway can be the main signaling pathway to govern the primordial follicle activation (Li et al., 2021; Vo and Kawamura, 2021). Interestingly, it has been shown that overexpression of CDK6 increased the activation of PI3K/AKT signaling pathway in primary mouse hippocampal neurons, suggesting the potential link between CDK6 expression and PI3K/AKT signaling pathway (Zhang et al., 2021). In line with this, CDK6 inhibition reduced the activity of MAP-ERK and PI3K/AKT/mTOR signaling pathways in an acute myeloid leukemia cell line (Nakatani et al., 2021). IN summary, these studies support our observations that inhibition of CDK6 could reduce the PI3K/AKT pathway, which would lead to reduce activation of primordial follicles. If note, and in contrast, it has been determined that CDK6 inhibition induced phosphorylation of AKT1S1 and increased the activation of mTORC1 signaling (Chang et al., 2022), but the details of how this is achieved is not known.

Our results showed that CDK6 inhibition increased apoptosis, particularly in granulosa cells, suggesting the possible effect of CDK6 inhibition on follicular or oocyte quality through induction of apoptosis. In line with this, it has been reported that immature *Cdk6*-deficient thymocytes show reducing in proliferation and induction of apoptosis (Hu et al., 2009). Moreover, the combination of CDK6 inhibition and a mitogen-activated protein kinase may result in cell cycle arrest and increasing in apoptosis a NRAS mutant melanoma mouse model (Kwong et al., 2012). Despite induction of apoptosis in this current study, CDK6 inhibition had no significant effect on ROS levels, which is known as one another marker for oocyte quality. In support of this, it was reported that CDK6 inhibition using Quercetin had no negative effect on viability of breast cancer and human adenocarcinoma cells, associating with induction of apoptosis (Yousuf et al., 2020). In contrast, it has been shown that CDK6 inhibition can impair antioxidants and significantly enhance ROS level (Chang et al., 2022). Moreover, it has been reported that CDK6 can regulate stabilization and activation of FOXM1, as a substrate of CDK4/6-Cyclin D complexes, leading to reduction of ROS production (Anders et al., 2011). IN summary, the effect of CDK6 inhibition might depend on the cell system used and also the specific functions of the cell population investigated.

These controversies guided this study to address the effect of CDK6 inhibition on oocyte quality, more extensively. Surprisingly, the *in vitro* cultured ovaries with CDK6 inhibitor had capability to produce competent *in vitro* matured MII oocytes, confirming no harmful effect of CDK6 inhibition on quality and meiotic maturation of oocytes. In consistent with our results, previous investigations on mice have determined that deletion of *Cdk6* could produce healthy animals with normal oocyte maturation, showing that *Cdk6* is not necessary for meiotic maturation of oocytes (Kohoutek et al., 2004; Adhikari et al., 2012; Risal et al., 2016). Moreover, Oqani et al. (2017) determined that inhibition of CDK6 by Palbociclib in porcine oocytes, did not affect cumulus expansion or formation of GVBD. Although, the *in vivo* functional role of CDKs during meiotic resumption of oocytes is still unravel, recent studies have shown that lacking of *Cdk6* in mice may be compensated by *Cdk1*, which can form active complexes with cyclins (Santamaria et al., 2007; Adhikari et al., 2012). In contrast, it has been reported that depletion of *Cdk6* in insects significantly reduces ploidy, arrested ovarian growth and oocyte maturation (Wu et al., 2018). Moreover, it has been shown that inhibition of CDK4/6 kinases in mice impaired spindle assembly, chromosome alignment and kinetochore-microtubule attachments following by increasing in progression of meiosis resulting in generation of aneuploid oocytes (Dong et al., 2021). However, there are many controversies between studies and the precise action of CDK6 during oocyte meiotic progression is still ambiguous, and awaits further investigations. In conclusion, the present study showed that CDK6 inhibition can regulate primordial follicle activation, with no effect on the quality and meiotic maturation of oocytes. However, the involved molecular mechanism of CDK6 inhibition in regulation of primordial to primary follicle transition is still unknown and requires further investigations.

## Material and methods

### Transcriptome data

The CDK6 gene expression data was extracted from two previous studies (Ernst et al., 2018, Ernst et al., 2017) using the large-scale data files http://users-birc.au.dk/biopv/published_data/ernst_2017 and http://users-birc.au.dk/biopv/published_data/ernst_et_al_GC_2017.

In these studies, the human ovarian cortical tissues were taken from three patients (26, 34 and 34 years old) undergoing oophorectomy before gonadotoxic treatment of a malignant disease. The patients normo-ovulatory, non-stimulated and with normal levels of reproductive hormones. In these global gene expression files FPKM values for all transcripts were calculated based on triplicate FPKM values using a one-sample *t*-test, based on a class comparison study of existing oocyte and granulosa cell transcriptomes from primordial (*n* = 539 follicles) and primary (*n* = 261) follicles collected from three patients. All transcripts were subsequently sorted according to their expression consistency across triplicates, as indicated by the one-sample *t*-test *p*-value (the cut-off value for inclusion in downstream analysis: *p* < 0.2 across triplicates). The CDK6 gene contribution in granulosa cells from primordial and primary follicles were collected and analysed using the global transcription lists (http://users-birc.au.dk/biopv/published_data/ernst_2017 and http://users-birc.au.dk/biopv/published_data/ernst_et_al_GC_2017) for 1) oocytes from primordial follicles 2) oocytes from primary follicles, 3) primordial follicles (oocytes with surrounding granulosa cells), and d) primary follicles (oocytes with surrounding flattened granulosa cells). Consistency in mean gene expression level (from the FPKM values) for all detected transcripts was quantified by performing a *t*-test on patient triplicate samples from oocytes and granulosa cells from primordial and primary follicles, respectively. Consistency was ranked based on *p*-value, with a low *p*-value indicating a high degree of consistency in the mean FPKM across patient triplicates.

### Animal

Female C57BL/6JRj mice and male CBA/JRj mice were housed under a 12-h light/dark cycle and breeding was performed to generate C57BL × CBA F1 mice in the biomedical animal facilities at Aarhus University. All procedures were approved by the Ethics Committee for the Use of Laboratory Animals at Aarhus University (permit number: 2020-15-0201-00757 to KLH).

### *In vitro* ovarian culture

The ovaries were separated from 7 to 8 days old mice after cervical dislocation. The ovaries were then isolated from their surrounding tissues and washed with alpha-minimal essential medium (α-MEM, Gibco, Scotland, United Kingdom) supplemented with 10% fetal bovine serum (FBS, Gibco). The collected ovaries were transferred to inserts (pore size of 0.4 mm, 6.5 mm diameter, Corning, MA, United States) which were placed in 24-well plates at 37 C and in a humidified atmosphere of 5% CO2–95% air. The ovaries were cultured in α-MEM supplemented with 10% FBS; 5 mg/ml insulin, 5 mg/ml transferrin, and 5 ng/ml sodium selenite (1% ITS; Gibco Cat. No. 41400045); 1% penicillin–streptomycin solution (Thermo Fisher Cat. No. 15140122); and 100 mIU/ml rFSH or GONALf (Serono) for 5 or 7 days. In addition, BSJ-03-123 (TOCRIS, Batch No: 1A/242335) at 1, 5, 10, 20, 30, 40, and 50 µM concentrations (reconstituted in DMSO) or only DMSO (DMSO control group) were administered to the culture medium, which were replaced with fresh culture medium every other day during culture period. The cultured ovaries for 5 days were collected for histological assessment, TUNEL assay, immunofluorescent staining, ROS assay and western blotting. The cultured ovaries for 7 days were considered for follicle culture.

### Histological assessment

*In vitro* cultured ovaries were fixed in paraformaldehyde 4% (PFA; Merck, 1.04005.1000) overnight at 4°C. Then, ovaries were dehydrated in a series of ethanol at room temperature (RT): 70%, 80%, 90%, 100% and the ovaries were cleared in xylene (VWR, 28.973.363) followed by infiltrating and embedding in paraffin at 60°C. The embedded tissues were then serially cut into 5 μM slices using Microtome (SLEE medical GmbH, cut6062) and stained by hematoxylin and eosin (H&E). For H&E staining, paraffin-embedded sections were deparaffinized at 60°C for 30 min followed by placing in xylene two times, 15 min in each. Next, the sections were rehydrated in a series of ethanol, placed in hematoxylin (Merck, 1.04302.01000) for 40 s, washed in water and transferred to eosin (Merck, 1.15935.0025) for 46 s, followed by dehydrating in a series of ethanol. The sections were then cleared in xylene and mounted using mounting medium (Sigma, 03,898). Follicle counting was conducted on every fifth section of entire ovaries. The follicles at different developmental stages were classified, previously. Briefly, the primordial follicles were defined as a single layer of flattened pre-granulosa cells surrounding an oocyte. The primary follicles were considered as a single layer of cuboidal granulosa cells surrounding an oocyte and the follicles contained two or more layers of granulosa cells were defined as secondary follicles. Only follicles containing a nucleus were counted to avoid recounting of the same follicle. An inverted microscope (Leica, BMI4000B) was used for assessment of the follicle stages.

### TUNEL assay

A TUNEL (TdT-mediated dUTP-X nick end labeling) assay was performed for detection andquantification of apoptosis within ovaries affected by increasing concentration of BSJ using Cell Death Detection Kit (Sigma, 11684795910), according to the instruction of manufacture with few adjustments. Briefly, the cultured ovaries for 5 days, were fixed, dehydrated, embedded, and sectioned as previously described. Then, the slides were deparaffinized and rehydrated followed by permeabilizing using proteinase K. Next, the labelled sections with TdT were incubated for 2 h in a humidity chamber at 37°C, while the negative controls were omitted the TdT. Subsequently, the sections were counterstained with DAPI. The sections were analyzed immediately using fluorescent imaging (Molecular devices, PICO imageXpress) by calculating the number of apoptotic cells compared to total number of cells in sections of ovarian cortex.

### Immunofluorescent staining

The immunofluorescence (IF) was performed for detecting intracellular proteins localization. The cultured ovaries were fixed, dehydrated, embedded and sectioned at 5 µm. The experiments were performed in three replicates and representative sections from the ovaries were included in each replicate. The slides with sections were deparaffinized, rehydrated and subjected to antigen retrieval with 0.01 M sodium citrate buffer (pH 6.0) at high temperature (95–98°C) for 15 min. The slides were then rinsed thoroughly with PBS, permeabilized using 0.5% Triton 100X for 10 min and blocked with normal donkey serum in PBS (10%) for 30 min at room temperature. Next, the slides were incubated with CDK6 (1:100, PA5-27978, Invitrogen) diluted in PBS containing 10% donkey serum, as primary antibody, overnight at 4°C. The next day, sections were washed in PBS followed by incubating with secondary antibody matching the serum block and primary antibody (Donkey Anti-Rabbit Alexa Flour 488 (1:300) for 1 h at RT. Then, the slides were washed in PBS, counterstained with DAPI for 3 min and mounted with fluorescence mounting medium (Dako, S3023). The sections were analyzed by an LSM510 laser-scanning confocal microscope using a ×63 C-Apochromat water immersion objective NA 1.2 (Carl Zeiss, Göttingen, Germany). The images were captured and assessed by Zen 2011 software (Carl Zeiss).

### Western blotting analysis

*In vitro* cultured ovaries were rinsed in PBS, pooled and lysed in RIPA buffer contained protease and phosphatase inhibitors. Concentration of protein was detected by the Lowry method with BSA quantification as a standard, and 20 µg was used per lane. Samples were then heated for 10 min at 70°C, before electrophoresis. Western blotting was performed using electrophoresis on SDS-PAGE gels and transferring onto PVDF membranes was performed for 1.5 h at 70 V. Membrane blocking was applied using Tris-buffered saline (pH = 7.6) supplemented with 0.1% Tween-20 and 5% non-fat dry milk for 1 h at room temperature. Next, the membranes were incubated overnight with CDK6 (1:500, PA5-27978, Invitrogen), Phospho-AKT (Ser473) (1:2000, Cell signaling technologies, 4060) and AKT (1:1000, Cell Signaling Technology, 9272) as primary antibody and beta-actin (1:3000, Sigma, A5441), as the loading control. The membranes were then washed with TBST (0.1% tween 20) followed by incubating with matching secondary antibody (1:3000, ThermoFisher Scientific, 65-6120 and 61-6520) for 1 h at room temperature. Visualization was performed using a chemiluminescence-based substrate and ImageJ was applied for quantification of the bands.

### ROS assay

The ovaries were washed in PBS and incubated with 40 mmol/L Tris–HCl buffer (pH = 7.0) containing 5 mmol/L DCFH-DA (Sigma) at 37°C for 30 min. The sample were rinsed again with PBS and lysed in 10 mM Tris-HCl containing 20 mM EDTA and 0.25% Triton 100X, followed by centrifuging at 4°C and 10,000 × *g* for 20 min. Finally, the supernatants were loaded into a black 96-well plate and assessed using a spectrofluorometer at 488 nm excitation and at 525 nm emissions.

### Encapsulation and three-dimensional *in vitro* culture of isolated secondary follicles

The ovaries were divided in two groups and cultured with BSJ at 5 µM concentration or DMSO for 7 days followed by isolation of secondary follicles (diameter between 60–82 µm) using insulin-gauge needles under a stereomicroscope (Leica, MZ75). The isolated follicles were then encapsulated using sodium alginate as previously described. Briefly, sodium alginate solution was prepared at a concentration of 0.5% (w/v) followed by filtering the solution. Next, each follicle was placed in a droplet of sodium alginate (7 µl), followed by transferring the droplets into a cross-linking solution (50 mM CaCl2 and 140 mM NaCl). The alginate beads were then washed using α-MEM medium. Finally, encapsulated follicles were cultured in basic α-MEM (with no BSJ or DMSO) supplemented with 10% FBS; 5 mg/ml insulin, 5 mg/ml transferrin, and 5 ng/ml sodium selenite (1% ITS; Gibco Cat. No. 41400045); 1% penicillin–streptomycin solution (Thermo Fisher Cat. No. 15140122); and 100 mIU/ml rFSH or GONALf (Serono) under mineral oil at 37 C with 5% CO2 for 12 days. Half of the culture media was changed with fresh media every other day, during culture period. Photomicrographs of the follicles were conducted using an inverted microscope (Leica, BMI4000B) at days 0, 6, and 12.

### *In vitro* ovulation induction

At the end of individual follicle culture period, antral follicles were mechanically released from sodium alginate beads using gauge 25 needles. Next, cumulus-enclosed oocytes (CEOs) were isolated from antral follicles released from sodium alginate beads, carefully, with no damage to the oocytes. Ovulation was then induced by transferring the CEOs to micro drops of α-MEM supplemented with 10% FBS; 5 mg/ml insulin, 5 mg/ml transferrin, and 5 ng/ml sodium selenite (1% ITS; Gibco Cat. No. 41400045); 1% penicillin–streptomycin solution (Thermo Fisher Cat. No. 15140122); 100 mIU/ml rFSH or GONALf (Serono) and 10 IU/ml hCG (Serono) under mineral oil at 37 C with 5% CO2 for 14 h.

### Statistical analysis

Data analysis was performed using GraphPad Prism software version 8 (GraphPad Software, San Diego, CA, United States). Data are shown as means ± standard error of the mean (SEM). Multiple group comparisons were performed using one way ANOVA followed by *post hoc* Tukey’s test. For all analyses, *p* values below .05 were considered to indicate statistically significant differences.

## Data Availability

The original contributions presented in the study are included in the article/Supplementary Material, further inquiries can be directed to the corresponding author.
