# Supplementary material for: Cyclin-dependent kinase 6 (CDK6) as a potent regulator of the ovarian primordial-to-primary follicle transition
Source: Front Cell Dev Biol. 2022 Dec 23;10:1036917. doi: 10.3389/fcell.2022.1036917 (PMC9816807; doi:10.3389/fcell.2022.1036917)
Supplement: Supplementary file 1 [file DataSheet2.PDF]

Supplementary data

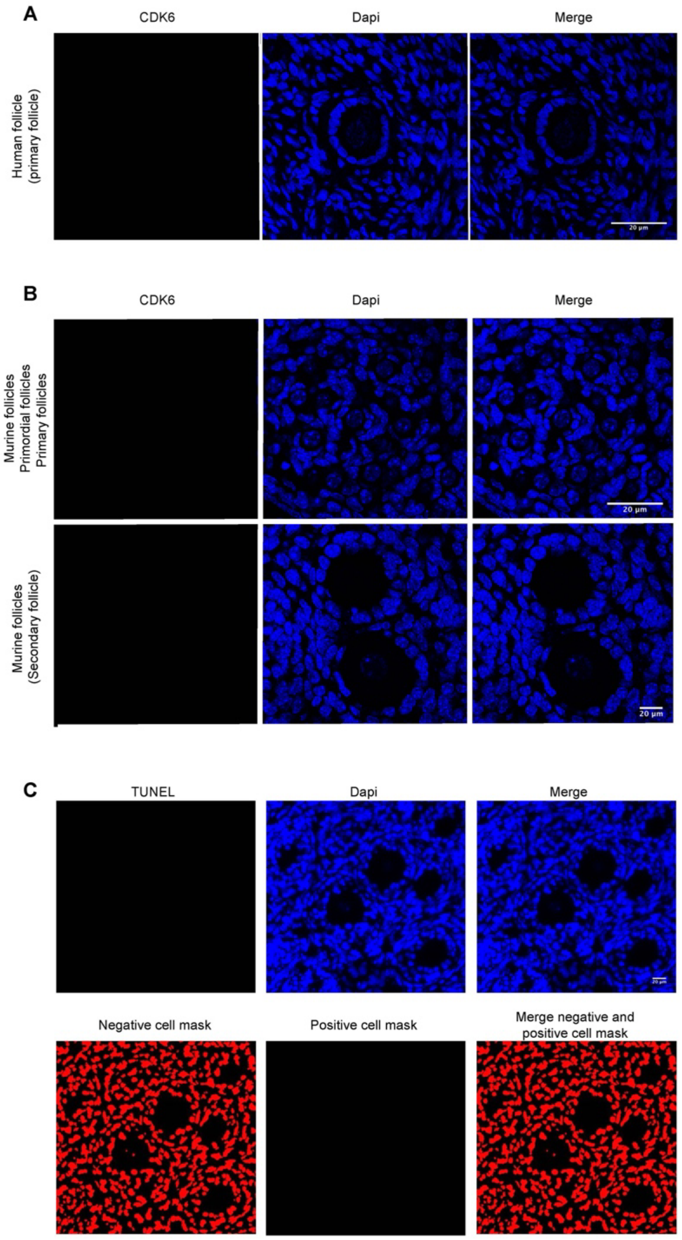

Figure S1. Negative controls.

(A) Negative immunofluorescence control omitting the primary antibody against CDK6 on human tissue. Scale bar: 20 µm. **(B)** Negative immunofluorescence control omitting the primary antibody against CDK6 on murine tissue with identified primordial, primary and secondary follicles. Scale bar: 20 µm. **(C)** Negative controls of TUNEL data omitting the reacting enzyme. Scalebar: 20 µm.

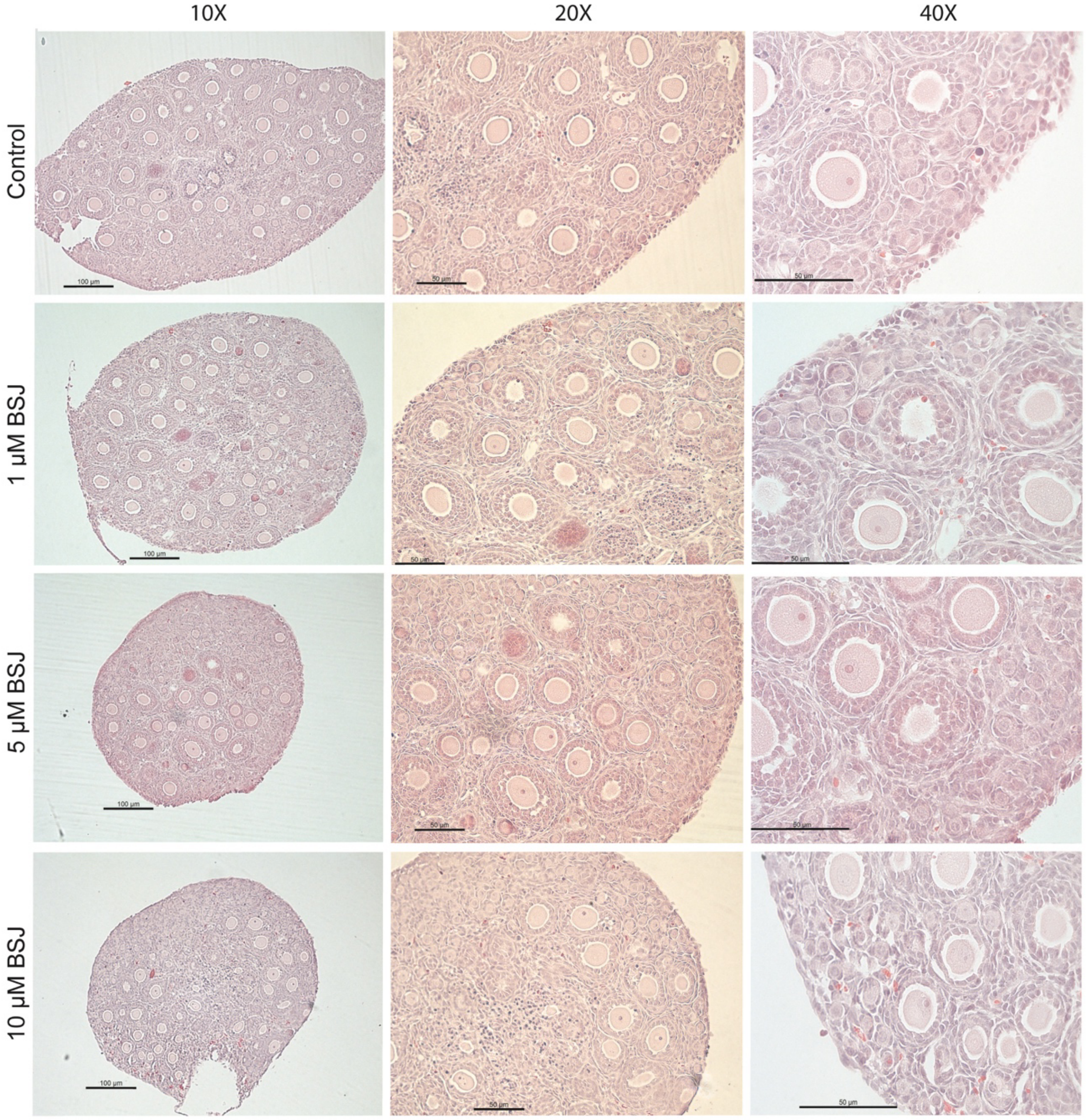

Figure S2. Photomicrographs of whole (10X) and representative sections (20X and 40X) of H&E stained ovaries exposed to different concentrations of BSJ (0-10 µM). Scalebar, first row: 100 µM and second and third row: 50 µM.

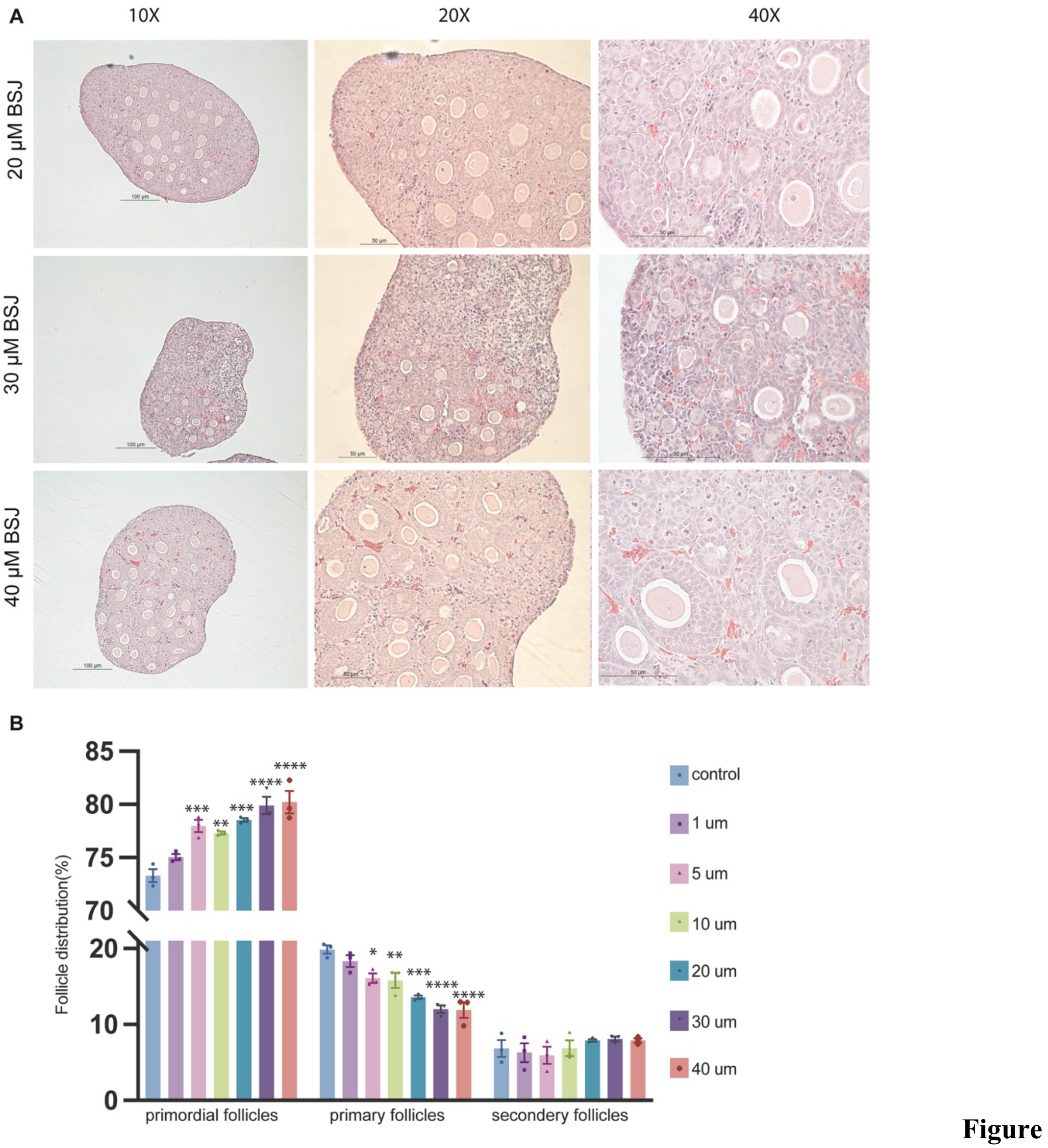

Figure S3. (A) Photomicrographs of whole (10X) and representative sections (20X and 40X) of H&E stained ovaries exposed to different concentrations of BSJ (20-40 µM). Scalebar, first row: 100 µM and second and third row: 50 µM. **(B)** The distribution of primordial, primary and secondary follicles in ovaries exposed to 0-40 µM BSJ. For all concentration *n*=3. The data is analyzed with one-way ANOVA followed by Bonferroni correction where the mean of each concentration is compared with the mean of the control. Statistically significant data are noted with asterisks, *$p < 0.05$, **$p<0.01$, ***$p<0.001$, ****$p<0.0001$.

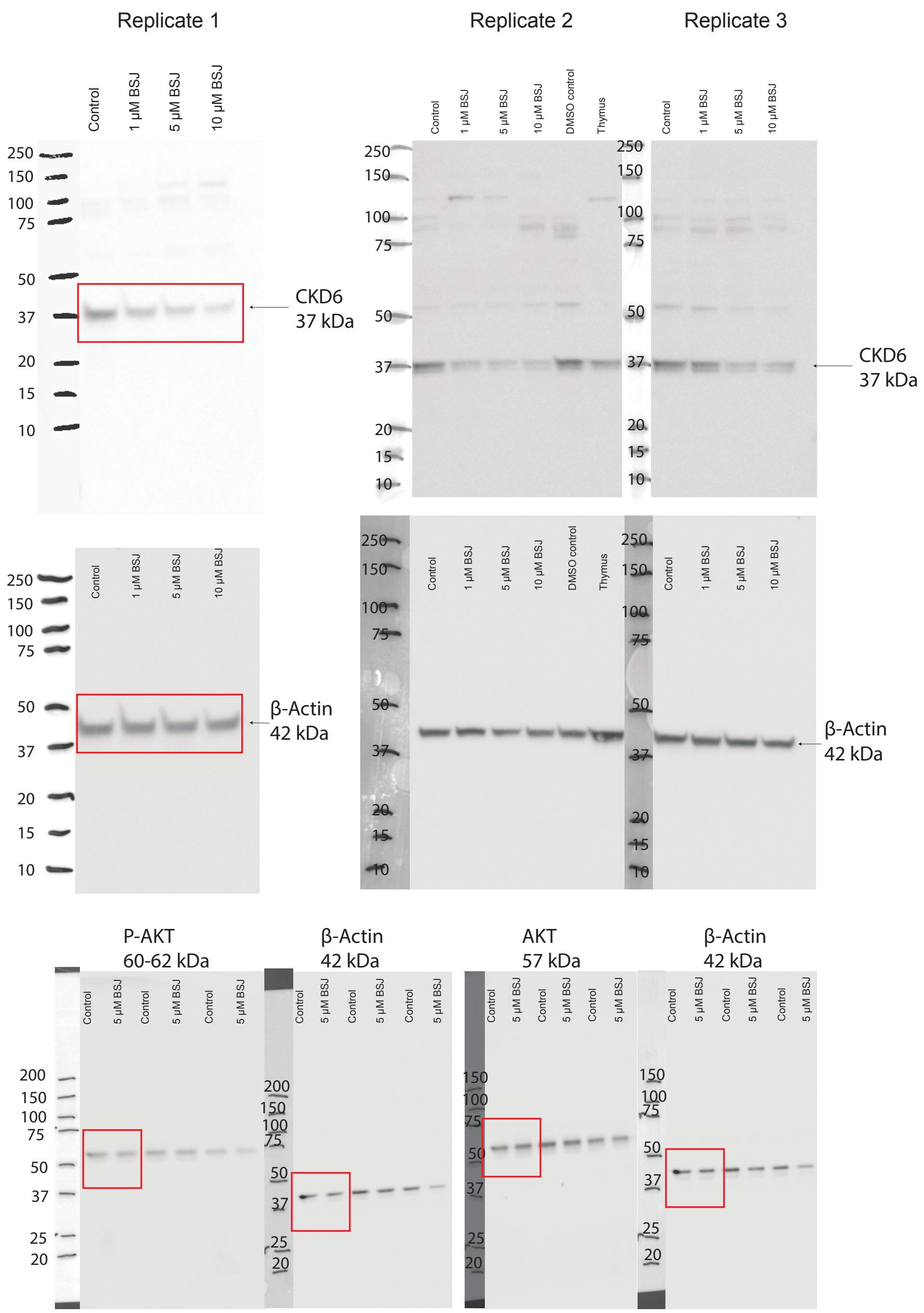

Figure S4. Full lengths Western blottings. Three independent replicates of membranes probed with CDK6, P-AKT, AKT and beta-actin as loading control. The red squares represent the bands in Figure 3. *n*=12.
